# HLA class I downregulation is associated with enhanced NK‐cell killing of melanoma cells with acquired drug resistance to BRAF inhibitors

**DOI:** 10.1002/eji.201445289

**Published:** 2015-12-20

**Authors:** Rosa Sottile, Pradeepa N. Pangigadde, Thomas Tan, Andrea Anichini, Francesco Sabbatino, Francesca Trecroci, Elvira Favoino, Laura Orgiano, James Roberts, Soldano Ferrone, Klas Kärre, Francesco Colucci, Ennio Carbone

**Affiliations:** ^1^ Department of MicrobiologyTumor and Cell BiologyKarolinska InstituteStockholmSweden; ^2^ University Magna Graecia of CatanzaroCatanzaroItaly; ^3^ Department of Obstetrics and GynaecologyUniversity of Cambridge School of Clinical MedicineNIHR Cambridge Biomedical Research CentreCambridgeUK; ^4^ Department of Experimental Oncology and Molecular MedicineFondazione IRCCS Istituto Nazionale dei TumoriMilanItaly; ^5^ Massachusetts General HospitalHarvard Medical SchoolBostonMAUSA

**Keywords:** acquired drug resistance, cytotoxicity, combination therapy, melanoma HLA, NK cell

## Abstract

The frequent development of drug resistance to targeted therapies in cancer patients has stimulated interest in strategies counteracting resistance. Combining immunotherapies with targeted therapies is one such strategy. In this context, we asked whether human NK cells can target melanoma cells that have acquired resistance to selective inhibitors targeting activating mutants of the B‐Raf kinase (BRAF inhibitors, BRAFi). We generated drug‐resistant cell variants in vitro from human *BRAF*‐mutant melanoma cell lines MEL‐HO, COLO‐38, SK‐MEL‐37, 1520 and from primary melanoma cells freshly isolated from two patients. All drug‐resistant cell variants remained susceptible to lysis by IL‐2‐activated NK cells; and two BRAFi‐resistant lines (BRAFi‐R) became significantly more susceptible to NK‐cell lysis than their parental lines. This was associated with significant HLA class I antigen downregulation and PD‐L1 upregulation on the drug‐resistant lines. Although blocking HLA class I enhanced the extent of lysis of both BRAFi‐R and parental cells to NK‐cell‐mediated lysis, antibody‐mediated inhibition of PD1–PD‐L1 interactions had no detectable effect. HLA class I antigen expression on BRAFi‐R melanoma variants thus appears to play a major role in their susceptibility to NK‐cell cytotoxicity. These findings suggest that NK‐cell‐based immunotherapy may be a viable approach to treat melanoma patients with acquired resistance to BRAF inhibitors.

## Introduction

Activation mutations in the serine‐threonine kinase B‐Raf are the most prevalent genetic lesions in malignant melanoma, with the V600E mutation in the *BRAF* gene being the most common 1. BRAF inhibitors (BRAFi), such as the *BRAF* ^V600E^–specific vemurafenib, also known as PLX4032, and dabrafenib, which targets *BRAF* ^V600E^ and also other mutant BRAF variants, significantly prolong overall survival of melanoma patients carrying these mutations 2, 3. Most patients treated with BRAFi, however, develop drug‐resistant tumors 4 and this is a major challenge.

New immunotherapies that target immune checkpoints, such as PD‐1 and its ligand PD‐L1, also offer survival gains to patients with metastatic melanoma, with durable response rates varying from 20 to 50% 5. Strategies are being tested to improve the response rates by combining immunotherapies with therapies that target mutant activated BRAF or its downstream pathway components 6, 7. For example, BRAFi enhance PD‐L1 expression on melanoma cells 8, providing an argument for combining therapies that target both BRAF and the PD‐1 pathway.

NK cells are innate immune cells that spontaneously lyse tumor cells, mediate antibody‐dependent cellular cytotoxicity and regulate adaptive immunity through production of cytokines. These functional properties make them an attractive target to co‐opt in immunotherapies for cancer patients. NK cells are activated by HLA class I molecule downregulation/loss on tumor cells, a frequent occurrence in cancer cells 9, 10. On the other hand, pharmacological agents that target cancer cells, such as dacarbazine 11, and P53 activators 12, 13, 14 induce the expression of stress‐induced molecules on melanoma cells that alert NK cells specifically through the NKG2D receptor.

The molecular mechanisms that regulate NK‐cell‐mediated recognition of cancer cells include both activating (NCRs, NKG2D, DNAM‐1, NKG2C) and inhibitory (KIR, NKG2A) receptors 15, 16, 17, 18. T‐cell inhibitory receptors PD‐1, CTLA‐4 and TIM‐3, which respectively bind PD‐L1/PD‐L2, B7‐1/B7‐2 and Galectin‐9, can also be induced in NK cells and regulate their function 19, 20, 21, 22. The effects of BRAFi on the crosstalk between melanoma cells and NK cells are unknown and here we set out to investigate them. We generated BRAFi‐resistant variants from four established BRAF^V600E^ and two freshly explanted human melanoma cell lines in vitro and measured: their susceptibility to lysis mediated by IL‐2 activated NK cells from healthy donors; expression of NK‐cell receptor ligands and HLA class I antigen processing machinery (APM) components in melanoma cells before and after acquisition of resistance to BRAFi vemurafenib or dabrafenib and effects of BRAFi on NK cells.

## Results

### Generation of BRAFi‐resistant melanoma cell lines

**Figure 1:**
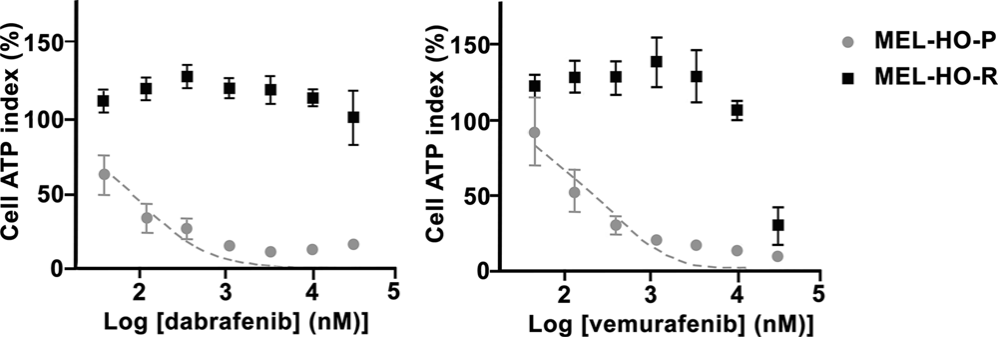
Effects of BRAFi on in vitro cell growth/viability of drug‐sensitive and ‐resistant melanoma cell lines harboring BRAF^V600E^. The drug‐sensitive and ‐resistant melanoma cell lines MEL‐HO and MEL‐HO‐R respectively were evaluated for sensitivity to BRAFi following 4 days culture in the presence of the indicated concentrations of dabrafenib (left) or vemurafenib (right). Cell growth/viability were determined by ATP assay (CellTiter‐Glo). Data are expressed as the mean luminescence ± SEM of the results pooled from three individual experiments.

We cultured the *BRAF^V600E^* mutant melanoma cell lines in the presence of BRAFi vemurafenib or dabrafenib at 5 μM for 30 days. Exposure to dabrafenib for at least 30 days lead to the emergence of a dabrafenib‐resistant variant of MEL‐HO cells (MEL‐HO‐R) that displayed increased resistance to dabrafrenib and cross‐resistance to vemurafenib, compared to the parental cell line (*p* < 0.0004) (Fig. 1). Similar experiments were done using other *BRAF^V600E^* mutant melanoma cell lines (COLO‐38, SK‐MEL‐37 and 1520, Table 1), and primary melanoma cell lines (Mel 30 and Mel 35, Table 1). The BRAFi‐resistant variants are thereafter referred to as MEL‐HO‐R, COLO‐38‐R, SK‐MEL‐37‐R, 1520‐R, Mel 30‐R and Mel 35‐R.

**Table 1:** Human melanoma cell lines used in this study

Cell line	BRAF status and other known mutations	References
MEL‐HO	*BRAF^V600E^, PTEN*	47
COLO‐38	*BRAF^V600E^*	48
SK‐MEL‐37	*BRAF^V600E^, PTEN, TP53*	49
1520	*BRAF^V600E^*	50
Mel 30	*BRAF^V600E^*	N.A.
Mel 35	*BRAF^V600E^*	N.A.

N.A.: not applicable

### Direct effects of BRAFi on NK cells

**Figure 2:**
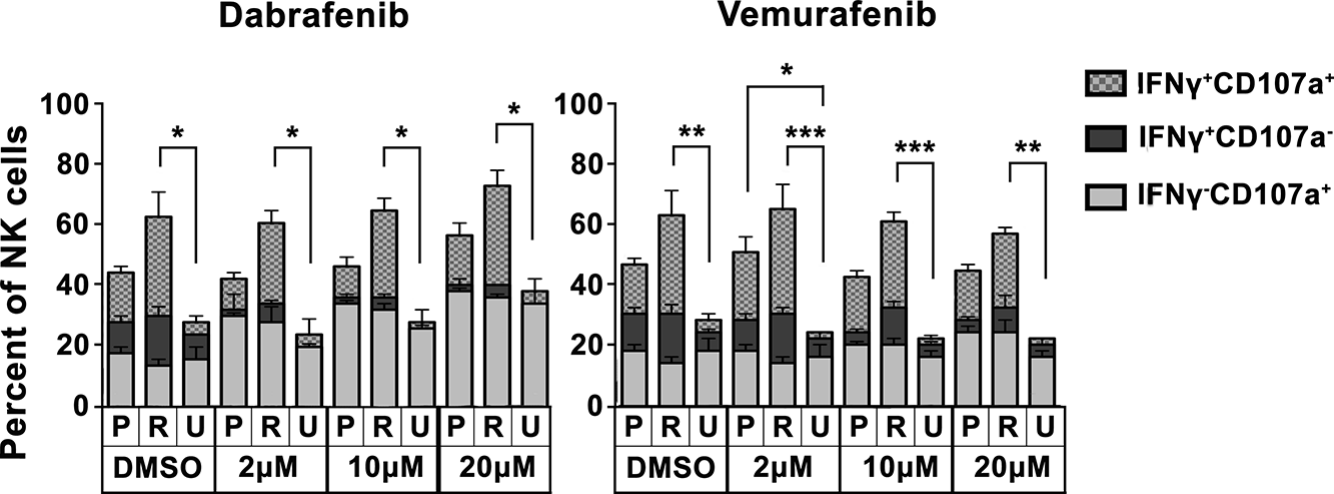
Effects of BRAFi on NK‐cell degranulation and cytokine response. Degranulation (CD107a) and cytokine (IFN‐γ) responses of BRAF‐treated, IL‐2‐activated NK cells were assessed by flow cytometry following 4 h in the absence (U) or presence of MEL‐HO‐P (P) or MEL‐HO‐R (R) target cells at 1:1 E:T ratio. Data are expressed as the geometric MFI ± SEM of the results pooled from three different donors. Aggregate values of IFN‐γ^+^CD107a^−^, IFN‐γ^−^CD107a^+^ and IFN‐γ^+^CD107a^+^ were compared by Bonferroni's multiple comparison test following 2‐way ANOVA test and the level of significance indicated as *p < 0.05, **p < 0.005, ***p < 0.005.

We next exposed IL‐2‐activated NK cells to BRAFi for 3 days in culture to assess the direct effects of BRAFi on NK cells. NK cells were obtained from three healthy donors. Following a 3‐day incubation with different doses of BRAFi, NK cells were co‐incubated with MEL‐HO or MEL‐HO‐R cells for 4 h after which the percentages of degranulating cells (CD107a^+^) and the level of produced IFN‐γ^+^ were quantified. The results show that neither vemurafenib nor dabrafenib reduced the frequency of responding NK cells, suggesting that the BRAFi tested here do not suppress NK‐cell effector functions. Moreover, we noticed that the MEL‐HO‐R cells induced a more potent NK‐cell response than the parental MEL‐HO cells (Fig. 2).

### NK cells lyse both BRAFi‐sensitive and BRAFi‐resistant melanoma cell lines

**Figure 3:**
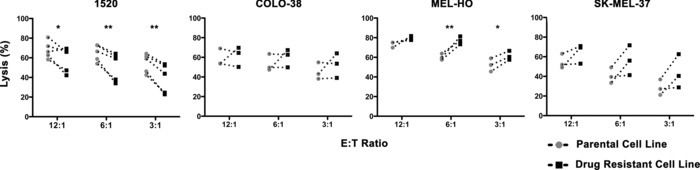
Susceptibility of both BRAFi‐sensitive and BRAFi‐resistant melanoma cells to NK‐cell‐mediated lysis. IL‐2‐activated NK cells were used as effectors and BRAFi‐sensitive (parental) and resistant (drug resistant) counterparts of the 1520, COLO‐38, MEL‐HO and SK‐MEL‐37 melanoma lines as targets in a standard chromium release assay. Data are expressed as percent lysis and are pooled from triplicates of at least three independent experiments. *p < 0.05, **p < 0.005 by Bonferroni's multiple comparison test following two‐way ANOVA test.

Allogeneic IL‐2 activated human NK cells from healthy donors were used as effectors against either the BRAFi‐resistant, and the respective parental, BRAFi‐sensitive melanoma cell line. IL‐2 activated donor NK cells lysed all four sets of BRAFi‐sensitive and BRAFi‐resistant cells (Fig. 3). 1520‐R cells were more resistant to NK‐cell‐mediated lysis than parental 1520 cells (*p* = 0.0397, 0.0044 and 0.0018 for E:T ratios 12:1, 6:1 and 3:1, respectively). In contrast, MEL‐HO‐R cells were more susceptible to NK‐cell‐mediated lysis than parental MEL‐HO cells (*p* = 0.0012 and 0.0234 for E:T ratios 6:1 and 3:1, respectively). No significant differences were detected in the killing rates of sensitive and resistant SK‐MEL‐37 and COLO‐38 cells.

### Immunomodulatory effects of BRAFi and HLA class I molecules on NK‐cell cytotoxicity

**Figure 4:**
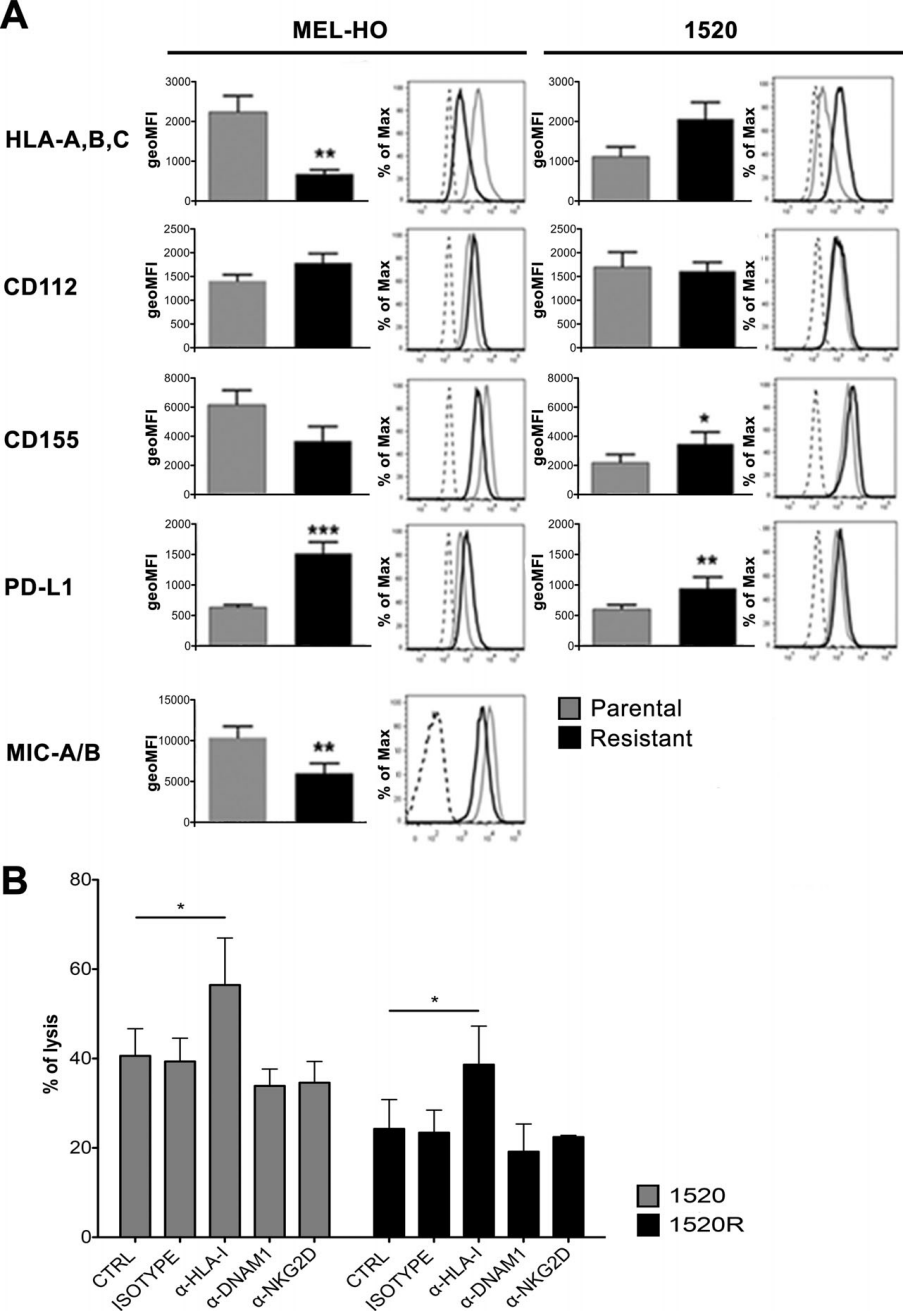
Differential expression of NK‐cell receptor ligands by BRAFi‐sensitive and BRAFi‐resistant melanoma cells and masking HLA class I molecules on BRAFi‐sensitive and ‐resistant cell lines enhances NK‐cell cytotoxicity. BRAF‐I resistance (resistant) was introduced to parental MEL‐HO and 1520 melanoma cell lines (parental). (A) Changes in cell surface expression of NK‐cell receptor ligands HLA‐A,B,C, CD112, CD155, PD‐L1 and MIC‐A/B upon acquisition of BRAFi resistance were determined by flow cytometry. Representative histograms are included for parental (gray) and resistant (black) MEL‐HO cells and parental (gray) and resistant (black) 1520 cells. Data are expressed as mean ± SD of the indicated numbers of samples/experiments. MelHO: HLA A,B,C *p = 0.0069*, PD‐L1 *p < 0.0001, n = 6*, MICA/B *p = 0.0033, n = 4* by paired Student's *t*‐test. 1520: PD‐L1 *p = 0.0092, n = 4* and of CD155 *p = 0.0192, n = 4* by paired Student's *t*‐test. **(B)** α‐HLA class I IgM antibody (clone A6/136) was used to mask HLA‐I molecules on BRAFi‐sensitive and BRAFi‐resistant 1520 cells. Melanoma cells were then exposed to IL‐2‐activated NK cells. To block NK‐cell receptors, IL‐2‐activated NK cells were pre‐incubated with the indicated blocking antibodies (α‐DNAM 1 mAb F5 IgM and α‐NKG2D mAb BAT221 IgG1) prior to coincubation with targets. The data are displayed as the mean ± SD of individual samples pooled from three independent experiments. *p < 0.05 by Bonferroni's multiple comparison test following two‐way ANOVA test.

We next quantified and compared HLA class I APM component and receptor ligand expression on 1520 and MEL‐HO cells as well as on 1520‐R and MEL‐HO‐R cells by flow cytometry analysis of cells stained with mAbs. The most interesting result was the significantly lower HLA class I antigen expression on MEL‐HO‐R cells as compared to the parental MEL‐HO cells (*p = 0.0069, n = 7*, Students’ paired *t*‐test, Fig. 4A). Analysis of of APM component expression in these two paired cell lines revealed that BRAFi‐resistant MEL‐HO‐R cells had downregulated HLA‐A and β2‐microglobulin (Supporting Information Fig. 1). Interestingly, 1520‐R cells, which were killed less effectively by NK cells than 1520 cells, displayed an upregulation of HLA class I molecule surface expression, although the difference between parental and resistant cells did not reach statistical significance (Fig. 4A). The expression of HLA class I molecules was comparable between the parental and resistant variants of COLO‐38, whereas the resistant variant of SK‐MEL‐37 cells slightly downregulated HLA class I molecules (data not shown).

To dissect the potential role of HLA class I molecules in controlling NK cells susceptibility of 1520R, HLA class I molecule masking was performed (Fig. 4B). The HLA class I‐specific blocking mAb restored susceptibility of these cells to NK‐cell‐mediated lysis, increasing their lysis up to the levels of the related parental BRAFi sensitive cells. On the other hand blocking activating receptors on NK cells had no detectable effect on the extent of killing between parental and resistant 1520 cell line. Our data clearly shows that NK‐cell inhibition by HLA class I molecules plays a role in the lower extent of lysis by NK cells of 1520R cells than of the parental 1520 cells.

MEL‐HO‐R cells displayed reduced expression of MICA/B (*p = 0.0033, n = 4)*, ligands of the activating receptor NKG2D, and increased expression of the PD‐1 ligand PD‐L1 *(p < 0.0001, n = 6)*. 1520‐R cells, compared to 1520 cells, displayed enhanced expression of PD‐L1 (*p = 0.0092, n = 4*) and of CD155 (*p = 0.0192, n = 4*), ligand of the inhibitory receptor TIGIT and of the activating receptor DNAM‐1 (Fig. 4A).

To define the potential role of PD1/PD‐L1 inhibitory interaction in the NK‐cell‐mediated lysis of melanoma BRAFi‐resistant variants anti PD1 antibodies were used to block the interaction of PD‐1 with its ligand PD‐L1. No major changes in the killing capacity of PD1 expressing NK cells against 1520‐R and MEL‐HO‐R were observed (Supporting Information Fig. 2). Therefore, it is conceivable that MEL‐HO‐R cells increased NK‐cell susceptibility is caused at least in part by their downregulated HLA class I antigen expression levels.

### NK‐cell cytotoxicity of short‐term BRAFi‐treated and established BRAFi‐resistant melanoma cells

**Figure 5:**
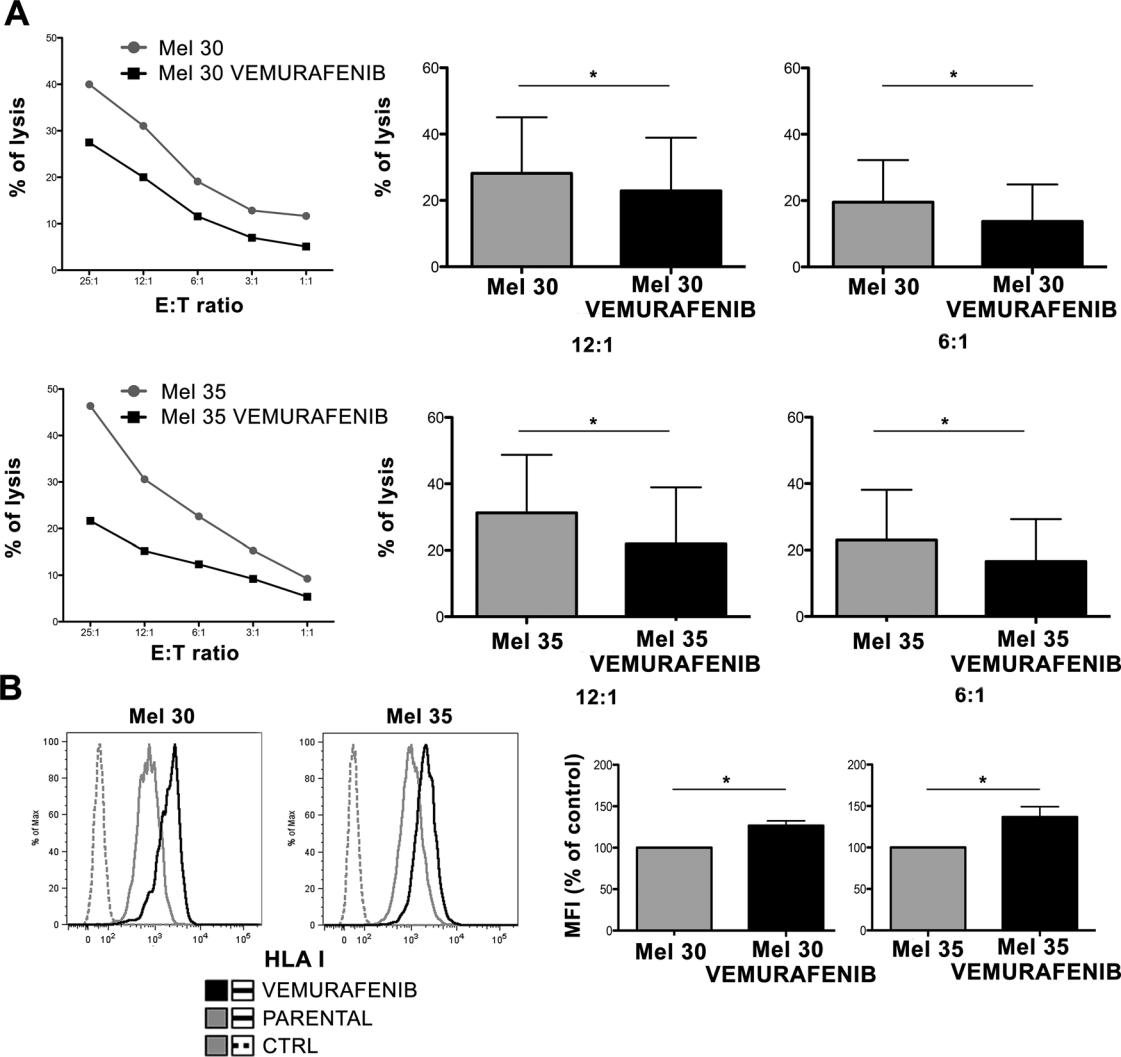
NK‐cell susceptibility of primary melanoma cells and surface expression of HLA class I antigens after short‐term treatment with vemurafenib. (A) Primary melanoma cells obtained from two different patients (Mel 30, top and Mel 35, bottom) were treated (or not) with vemurafenib for 24 h and then exposed to NK cells at the indicated E:T ratios. NK‐cell recognition of these melanoma lines was measured by specific lysis. A representative of three individual experiments showing the lysis of Mel 30 and Mel 35 (left) and statistical significance between the killing of Mel 30, Mel 35 parental and Mel 30, Mel 35 vemurafenib at two different indicated E:T ratios (right) are shown. Data are expressed as the mean ± SD of triplicates pooled from three independent experiments. Mel 30 12:1 *p* = 0.0128 and 6:1 *p* = 0.0476, Mel 35 12:1 *p* = 0.0322 and 6:1 *p* = 0.0457 paired Student's *t*‐test. **(B)** Primary melanoma cells (Mel 30 and Mel 35) were treated with vemurafenib for 24 h and the cells were then stained with mAb W6/32 directed against HLA A, B, C. Representative histograms for Mel 30 and Mel 35 where parental (gray), treated with vemurafenib (black) and isotype (dashed) are included. The mean ± SD of individual samples pooled from three independent experiments (right) is also shown. Mel 30 *p* = 0.0153, Mel35 *p* = 0.0371, paired Student's *t*‐test.

Using freshly established primary melanoma cells, we evaluated the effect of BRAFi on their NK‐cell susceptibility after short‐term drug exposure. Mel 30 and Mel 35 cells were treated with vemurafenib for 24 h prior to be exposed to NK cells. Vemurafenib treated cells appear to be significantly less susceptible to NK‐cell killing than the parental cells (Mel 30 12:1 *p* = 0.0128 and 6:1 *p* = 0.0476, Mel 35 12:1 *p* = 0.0322 and 6:1 *p* = 0.0457, Fig. **5** **A**). This difference may be caused at least in part by the significant upregulation of HLA class I antigen expression on the vemurafenib treated Mel 30 and Mel 35 cells (Mel 30 *p* = 0.0153, Mel 35 *p* = 0.0371, Fig. 5B). Similar results were obtained using dabrafenib (Supporting Information Fig. 3).

Additional evidence in support of the role of the increased HLA class I expression in the resistance of the short‐term BRAFi‐treated primary melanoma cells to NK cells mediated killing, comes from the lack of any significant changes in the surface expression of other NK‐cell ligands on both Mel 30 and Mel 35 (Supporting Information Fig. 4).

**Figure 6:**
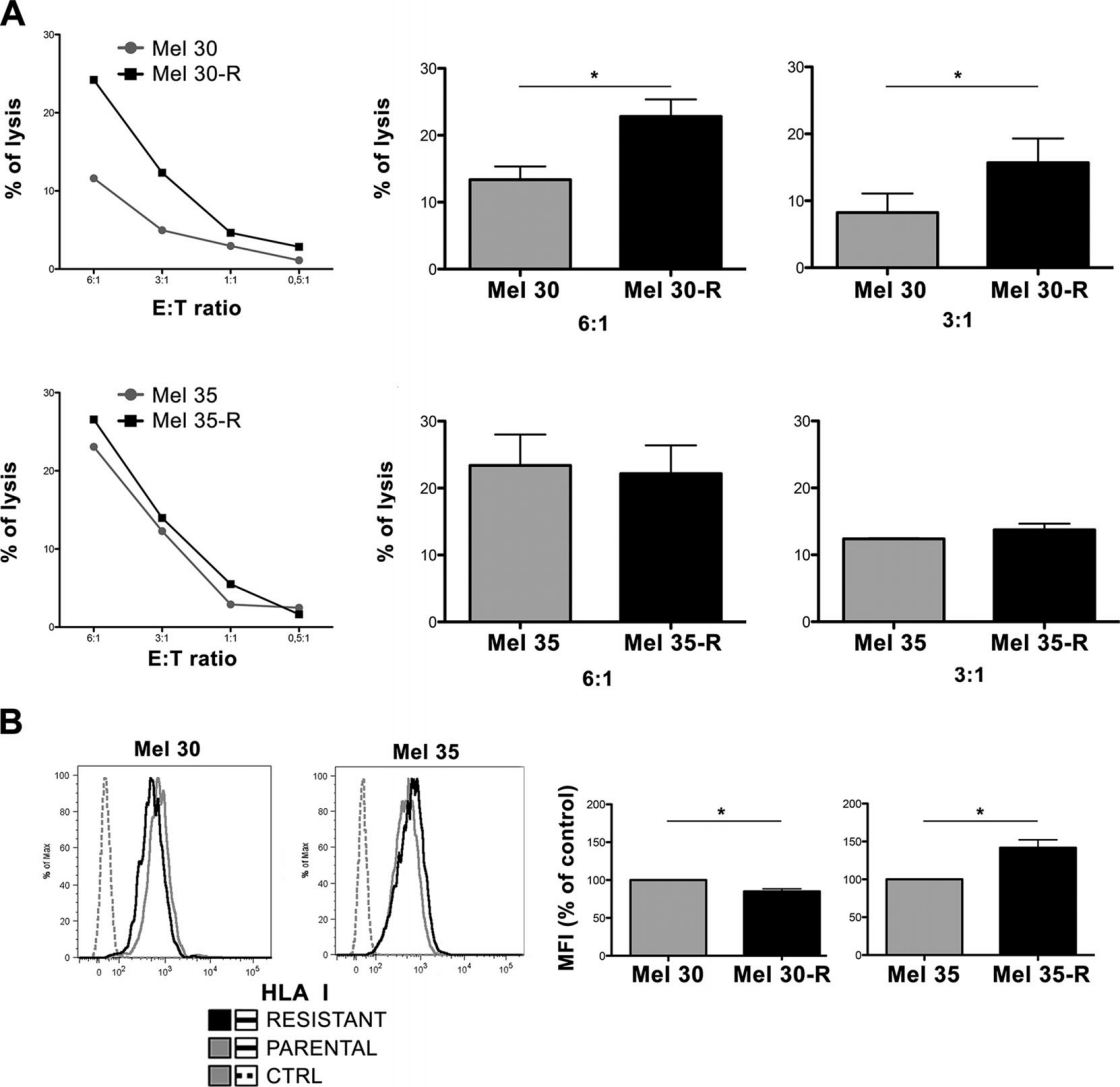
Susceptibility to NK‐cell cytotoxicity and HLA class I surface expression of BRAFi‐resistant primary melanoma cells. (A) Primary melanoma cells collected from two patients (Mel 30, top and Mel 35, bottom) were cultured for 4 weeks with vemurafenib and then exposed to NK cells at the indicated E:T ratios. NK‐cell recognition was measured by specific lysis.. A representative of three independent experiments showing specific lysis of Mel 30 and Mel 35 (left), and statistical significance between the killing of Mel 30, Mel 35 parental and Mel 30, Mel 35 resistant cells (right) are shown. Data are expressed as the mean ± SD of triplicates samples pooled from three independent experiments. Mel 30 6:1 *p* = 0.0303, 3:1 *p* = 0.0256, paired Student's *t*‐test. **(B)** Primary melanoma cells (Mel 30 and Mel 35) were treated with vemurafenib for 4 weeks and the cells were then stained with HLA class I antibody W6/32. Representative histograms for Mel 30 and Mel 35 where parental (gray) and resistant (black) and isotype (dashed) are included (left). Data are also expressed as the mean ± SD of individual samples pooled from three independent experiments., Mel 30 *p* = 0.0159, Mel 35 *p* = 0.0203, paired Student's *t*‐test.

Vemurafenib‐resistant variants were subsequently generated from Mel 30 and Mel 35 parental cells and NK cells susceptibility was measured. With the long‐term treatment of vemurafenib, Mel 30‐R cells became significantly more susceptible to NK‐cell killing than their parental cell (6:1 *p* = 0.0303, 3:1 *p* = 0.0256, Fig. 6A). In contrast, Mel 35‐R cells showed at most a slightly reduced susceptibility to NK‐cell recognition, as compared to their parental cells. A reduction of surface expression of HLA class I was again observed in BRAFi‐resistant Mel 30‐R cells. While vemurafenib resistant Mel 30‐R cells expressed less HLA class I compared to related parental cell (*p* = 0.0159, Fig. 6B), vemurafenib resistant Mel 35‐R cells expressed a significantly higher level of HLA class I antigens than their parental cells (*p* = 0.0203 Fig. 6B). Therefore, the results observed with the primary melanoma samples Mel 30 and Mel 35 recapitulate the patterns obtained with the cell lines after the generation of the resistant variants. Mel 30 resembles MEL‐HO both in terms of susceptibility to NK‐cell killing and in terms of HLA class I antigen surface expression, while Mel 35 cells resemble 1520 cells in terms of the increased HLA class I antigen surface expression on the BRAFi‐resistant cells.

Interestingly, as observed with MEL‐HO‐R and 1520‐R cells, Mel 30‐R and Mel 35‐R cells express detectable levels of the PD‐1 ligand (PD‐L1) in a higher proportion of cells, as compared to that measured in the related BRAFi‐sensitive cells (*p* value = 0.0042 and 0.0006, respectively, Supporting Information Fig. 5). It should be noted that there is no correlation between the expression levels of PD‐L1 on Mel 30, and Mel 35 cells and their corresponding variants and their susceptibility to NK‐cell‐mediated lysis.

## Discussion

We provide evidence that NK cells are able to lyse melanoma cell lines that have acquired resistance to BRAFi and BRAFi do not suppress NK‐cell effector functions. These results provide arguments to consider harnessing NK‐cell‐mediated cytotoxicity to improve the success of therapies in melanoma patients harboring mutant BRAF, and in particular those who have become refractory to BRAFi treatment. Acquisition of BRAFi resistance is associated with decreased HLA class I antigen expression in the case of the MEL‐HO cell line and in less extent in Mel 30 primary cells while an increase of the same is clearly observed in 1520 cell line and Mel 35 primary cells. A large screening of the melanoma cell lines surfaces searching for molecules known to modulate NK cells susceptibility leads to the appreciation of the HLA class I antigens as potential regulatory molecule for the NK‐BRAFi‐resistant melanoma cross talk. The upregulation of HLA class I antigens contribute to the drug‐resistant cells reduced susceptibility to NK‐cell cytotoxicity. Masking of HLA class I molecules results in restored susceptibility of 1520R cells to the levels of parental 1520 cells. It is tempting to speculate that HLA class I APM molecule downregulation in MEL‐HO‐R cells might have led to engagement of fewer inhibitory KIRs in NK cells. This in turn might render drug‐resistant MEL‐HO‐R cells more susceptible to NK‐cell‐mediated lysis. On the other hand, acquisition of resistance to BRAFi in 1520 cells correlated with a tendency to upregulate HLA class I APM molecules and a reduced ability of NK cells to kill the BRAFi‐resistant variant. The HLA class I masking experiment performed on 1520 cell pairs results in an increased NK susceptibility of BRAFi‐resistant and related susceptible melanoma cells suggesting that biological therapy interfering with the HLA‐KIR checkpoint may be an effective strategy to target drug‐resistant melanoma cells for destruction by NK cells. However, it should be noted that in our experiments the use of a pan anti‐HLA class I monoclonal antibody could underestimate the surface expression levels changes of either specific classical HLA class I alleles (HLA‐C, HLA‐B) or nonclassical HLA class I molecules (HLA‐E, HLA‐G). Therefore, this part of the study deserves a more detailed analysis at higher resolution.

The expression of PD‐L1 was enhanced on MEL‐HO‐R and 1520‐R, as well as in Mel 30‐R and Mel 35‐R confirming previous work showing that activation of MAPK, which is a major mechanism of acquired BRAFi resistance, promotes PD‐L1 expression 8. PD‐L1 exerts its immunosuppressive role mainly through engaging PD‐1 on activated T cells, however there are precedents for potential roles of PD‐1 in NK‐cell function. Indeed, the expression of PD‐1 on NK cells may predict responses to anti‐PD1/PD‐L1 therapies in patients with multiple myeloma 19. PD‐1 expression on blood leukocytes, including NK cells, could be a useful indicator of disease progression in patients with renal cell carcinoma 23. However, our results of the blocking experiments with PD‐L1‐specific mAbs argue against an involvement of this molecule PD‐L1 in NK‐cell recognition of the BRAFi‐resistant variants. BRAFi have been shown to exert immunomodulatory effects, such as upregulation of melanoma antigens in vitro 24, increased T‐cell infiltration in tumors 25, reversed immunosuppression by myeloid‐derived suppressor cells 26 and enhanced PD‐L1 expression 8. Vemurafenib causes changes in the proportions of peripheral blood lymphocytes, with decreased CD4+ T cells and increased NK cells 27. In a mouse model, a vemurafenib analog downmodulated expression of CCL2 on melanoma cells, resulting in increased intratumor CD8^+^ T cell/Treg ratio and enhanced proportion of NK cells 28, 29. Our results suggest that susceptibility to NK‐cell‐mediated lysis is preserved despite the acquisition of BRAFi resistance. Based on these results, we propose that IL‐2 activated allogeneic NK cells could be added to the arsenal of immunotherapies to be combined with targeted therapies.

The evidence that NK cells participate in tumor immunity is clear. Selective NK‐cell immunodeficiencies, albeit rare, are characterised by high prevalence of malignancy. Among 21 cases described, six developed malignancies, including one case of pediatric melanoma, one sarcoma and four hematological malignancies 30. An 11‐year longitudinal study in a large cohort showed increased cancer prevalence in individuals with a low NK‐cell cytotoxicity in peripheral blood 31. NK‐cell‐deficient mice fail to reject transplanted tumors 32. NK cells are being tested in several clinical trials that aim to either harness host NK cells or use donor NK cells in adoptive cell therapy 33. NK cells, together with CTL, mediate the effect of high dose IL‐2 treatment reported in early clinical trials in patients with metastatic cancers 34. Moreover, increased frequency of CD56^dim^, KIR^+^CD57^+^ highly cytotoxic NK cells in melanoma infiltrated lymph nodes correlate with patient clinical outcome 35. The results of our study provide further arguments in favor of considering adoptive NK‐cell therapy in melanoma patients that develop resistance to BRAFi.

## Materials and methods

### Cell lines and primary cells

All melanoma cell lines were maintained in RPMI 1640 medium supplemented with 10% heat inactivated fetal calf serum (FCS), 1% penicillin/streptomycin and 50 μM 2‐marcapto ethanol. Cells were passaged every 2–3 days. Primary melanoma cell lines carrying the *BRAF^V600E^* mutation were obtained from patients after informed consent, according to previously described procedure 36 at the Fondazione IRCCS Istituto Nazionale dei Tumori, Milan, Italy. Melanoma patients were named Mel 30 and Mel 35. Primary blood NK cells were isolated by negative selection with immunomagnetic beads (Miltenyi) according to manufacturer's protocol. Isolated NK cells were maintained in minimal essential medium with alpha‐modification supplemented by 10% heat inactivated FCS, 2nM L‐Glutamine and 1000 IU/mL IL‐2 Proleukin (Novartis, Basel) for 48 h.

### Antibodies and protein reagents

The following antibodies for flow cytometry were purchased from BioLegend unless otherwise stated: anti‐CD3 (clone UCHT1) APC‐Cy7, anti‐CD56 [clone HCD56] Brilliant Violet 605, anti‐CD112 (clone TX31) R‐phycoerythrin (PE), anti‐CD155 (clone TX24) PE, anti‐CD274 (PD‐L1, clone 29E.2A3) PE or anti‐CD274 (PD‐L1, clone 10F.9G2) PE, anti‐HLA‐A, B, C (clone W6/32) PE, anti‐MIC‐A/B (clone 6D4) Alexa Fluor 647, anti‐CD107a (clone H4A3) Alexa Fluor 647, anti‐IFN‐γ (clone 4S.B3) PE (from eBioscience). Antibody specific for HLA class I APM components include: HLA‐A‐specific mAb LGIII‐147.4.1, HLA‐B, C‐specific mAb B1.23.2, HLA‐A, B, C‐specific mAb TP25.99.8.4, β2‐microglobulin‐specific mAb NAMB‐1, calnexin‐specific mAb TO‐5, calreticulin‐specific mAb TO‐11 and tapasin‐specific mAb TO‐3, HLA‐DR, DQ, DP‐specific mAb LGII‐612.14. Cells were intracellularly stained with Delta‐specific mAb SY‐5, LMP2‐specific mAb SY‐1, LMP7‐specific mAb HB2, LMP10‐specific mAb TO‐7, TAP1‐specific mAb NOB1, TAP2‐specific mAb NOB2, calnexin‐specific mAb TO‐5, calreticulin‐specific mAb TO‐11 and tapasin‐specific mAb TO‐3 37, 38, 39, 40, 41, 42, 43, 44. The isotype matched IgG1 mAb MK2‐23 was used as a specificity control. Cell staining was detected by PE‐labeled anti‐mouse IgG antibody. mAb BAM 195 (anti‐MICA, IgG1) and mAb 6D4 (anti‐MICB, IgG1) were provided by Veronika Groh (Fred Hutchinson Cancer Research Center, Seattle, WA, USA); M295 (anti‐ULBP1, IgG1), M310 (anti‐ULBP2, IgG1), M550 (anti‐ULBP3, IgG3) and M478 (anti‐ULBP4, IgG1) were gifted by D. Cosman (Amgen, Seattle, WA, USA; mAb L95 (anti‐CD155, IgG1) and mAb L14 (anti–CD112, IgG2a) were developed and characterized as described in Bottino et al. 45. Cell staining was detected by PE‐labeled anti‐mouse IgG antibody. For the masking of HLA class I and blocking NK cell receptors the following antibodies were used: anti‐HLA‐A,B,C (clone A6/136, IgM), anti‐DNAM‐1 (clone F5, IgM), anti‐NKG2D (clone BAT221, IgG), anti‐CD‐57 (clone HNK‐1, IgM), all kindly provided from Alessandro Moretta (Laboratory of Molecular Immunology, Department of Experimental Medicine, University of Genova, Italy) and anti‐PD1 (clone EH12.2H7, LEAF^TM^ purified IgG1, Biolegend).

### Generation of BRAFi‐resistant cells

Melanoma cell lines with acquired BRAFi resistance (COLO‐38‐R, SK‐MEL‐37‐R, 1520‐R, MEL‐HO‐R) were generated by propagating parental COLO‐38, SK‐MEL‐37, 1520 and MEL‐HO cells in increasing concentrations of BRAFi (up to 5 μM). MEL‐HO cells in the presence of 5 μM vemurafenib failed to produce BRAFi‐resistant cells, whereas 30 days of culture in the presence of 5 μM dabrafenib gave rise to MEL‐HO cells with increased resistance toward dabrafenib and cross‐resistance to vemurafenib. At the end of cell culture period that induced resistance, resistant cells were isolated from each of the cell lines and cultured in RPMI 1640 medium supplemented with 2 mmol/L L‐glutamine, 10% FCS and 500 nM BRAFi. Prior to cytotoxicity assays and to flow cytometry, both sensitive and resistant cells were cultured in BRAFi‐free medium for up to 2 weeks. To check the short‐term effect of the drugs on primary cells, Mel 30 and Mel 35 were treated for 24 h before the experiment by 5 μM of either vemurafenib or dabrafenib. For the generation of Mel 30‐R and Mel 35‐R the relative parental cells were propagated for 30 days in the presence of 5 μM vemurafenib and after the appearance of the resistant cells the concentration of BRAFi was kept at 500 nM. Cytotoxicity and flow cytometry experiments were performed without removing the drug from the culture medium. We gene sequenced the resistant cell variants 1520‐R and MEL‐HO‐R to confirm that they had conserved the mutant *BRAF^V600E^* genotype and were not an outgrowth of potentially pre‐existing BRAF^WT^ cells (Supporting Information Fig. 6A). The BRAF^WT^ MV3 cell line was used as control. Western blots showed that SK‐MEL‐37‐R and COLO‐38‐R cells, but not 1520‐R cells or MEL‐HO‐R cells, had a truncated version of BRAF, which no longer binds BRAFi (Supporting Information Fig. 6B and not shown), and is known to confer resistance in about 30% of patients progressing on BRAFi therapy 46. Therefore it is likely that SK‐MEL‐37‐R and COLO‐38‐R cells acquire resistance due to the truncated BRAF. The mechanism of resistance to BRAFi in 1520‐R and in MEL‐HO‐R cells are unknown.

### Cell ATP luminescence assay

Melanoma cells were cultured in tissue culture treated 96‐well plates in the presence of various concentrations of dabrafenib, vemurafenib or DMSO for 4 days at 37°C and 5% CO_2_. Relative cell ATP levels were assayed by CellTiter‐Glo homogenous luminescence assay (Promega) according to manufacturer's protocol and luminescence was detected on a BMG OPTMA microplate reader.

### Cytotoxicity assay

Melanoma target cells were labeled with Na[^51^Cr]O_4_ at 37°C for 1 h and co‐incubated with NK effector cells in complete medium at 37°C for 4 h. The ^51^Cr activity of the supernatant was measured with a gamma‐counter. Percent specific lysis was calculated as ([cpm experimental release − cpm spontaneous release]/[cpm maximum release − cpm spontaneous release]) × 100. Spontaneous release represents the ^51^Cr release from target cells alone. Experimental release represents the release from target cells incubated with effector cells and maximum release represents the ^51^Cr content of resuspended target cells.

### Flow cytometry

Melanoma cells were detached nonenzymatically by 1× PBS with 2 mM EDTA. Dead cells were excluded by Hoechst viability stain 0.5 μg/mL. For cell surface staining, cells were blocked in Human TruStain FcX reagent (BioLegend) prior to staining with fluorochrome conjugated mAb and 7‐AAD viability stain followed by one wash in FACS‐buffer. For the degranulation and cytokine response assay, melanoma cells were labeled with DiO tracer dye (Life Technologies) according to manufacturer's protocol and cocultured with NK cells for 4 h at 37°C, 5% CO_2_ in the presence of 1:100 anti‐CD107a antibody, 5 μM monensin and 3 μg/mL brefeldin A. Following coculture, cells were stained with antibodies to cell surface markers and fixable viability dye (eBioscience). Intracellular IFN‐γ was detected by anti‐IFN‐γ antibody (eBioscience) according to manufacturer's protocol. All cells were acquired with a BD LSR‐Fortessa flow cytometer (BD Biosciences). Flow cytometry data were analyzed by FlowJo Software (TreeStar). Representative flow cytometry gating diagrams are shown in Supporting Information Fig. 7.

### Western blot assay

Adherent melanoma cells were lysed in RIPA buffer (Thermo Scientific) supplemented with Complete Proteinase Inhibitor tablets (Roche) and quantified by bicinchoninic acid whole protein assay prior to SDS‐PAGE separation under reducing conditions. Separated proteins were transferred to a PVDF membrane via the iBlot dry blotting system (Life Technologies) and blocked in 10% cleared skimmed milk followed by detection with rabbit‐anti‐BRAF mAb clone 55C6 (Cell Signaling Technology). Equal protein loading was confirmed by membrane stripping and reprobing with anti‐Actin antibody (Cell Signaling Technology).

### Statistical analysis

Two‐tailed Student's *t*‐test, one sample *t‐*test and two‐way parametric analysis of variance (ANOVA) with Bonferroni multiple‐comparison test were used to determine statistical significance; in these tests a *p* value of less than 0.05 was considered significant. Tests were performed with GraphPad Prism version 5.03 and 6.00 (GraphPad Software).

## Authors’ contribution

R.S., P.P., T.T., F.S., E.C. and F.C. conceived and designed experiments. R.S., P.P., T.T., F.S., E.F., L.O. and J.R. performed experiments and statistical analysis. E.C. and F.C. wrote the manuscript. R.S., P.P. and T.T. helped with editing the manuscript. S.F., E.C. and F.C. supervised the analysis and edited the manuscript. A.A. and K.K. provided insights.

## Conflict of interest

The authors declare no financial or commercial conflict of interest.

**Abbreviations**
BRAF: B‐Raf (Rapidly Accelerated Fibrosarcoma) proto‐oncogene
BRAFi: inhibitors of mutant B‐Raf kinase

## Supporting information

As a service to our authors and readers, this journal provides supporting information supplied by the authors. Such materials are peer reviewed and may be re‐organized for online delivery, but are not copy‐edited or typeset. Technical support issues arising from supporting information (other than missing files) should be addressed to the authors.

supporting information figure 1

Peer review correspondence
