# Supplementary material for: HLA class I downregulation is associated with enhanced NK‐cell killing of melanoma cells with acquired drug resistance to BRAF inhibitors
Source: Eur J Immunol. 2015 Dec 20;46(2):409–19. doi: 10.1002/eji.201445289 (PMC4832274; doi:10.1002/eji.201445289)
Supplement: Supplementary file 1 — supporting information figure 1 [file EJI-46-409-s001.pdf]

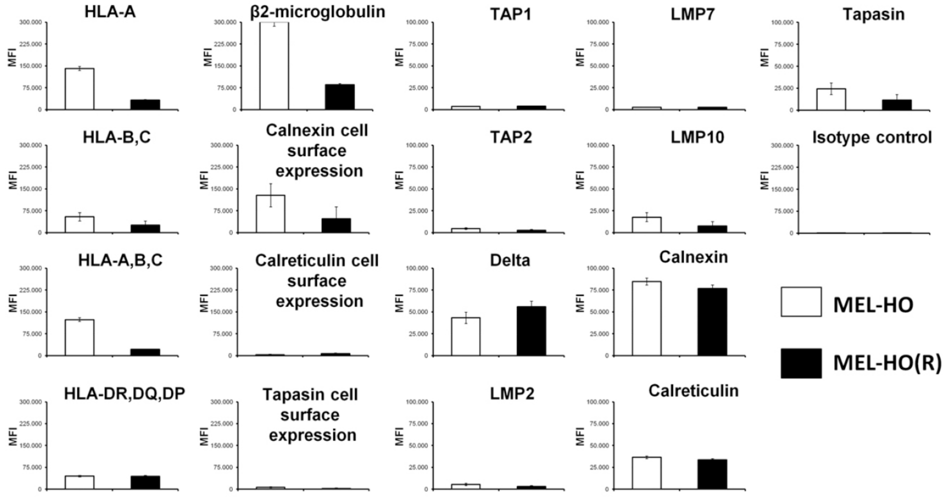

Supporting Information figure 1. HLA class I Antigen Processing Machinery component expression in BRAFi sensitive and resitant MEL-HO cells harboring BRAFV600E

Melanoma cells were acquired by FACS following cell surface staining with mAbs specific for HLA-A, HLA-B and C, HLA-A, B, C, β2-microglobulin, calnexin, calreticulin, tapasin, and and HLA-DR,DP,DQ. Cells were intracellularly stained with mAbs specific for Delta, LMP2, LMP7, LMP10, TAP1, TAP2, calnexin, calreticulin and tapasin. The isotype matched IgG1 mAb MK2-23 was used as a specific control. Cell staining was detected by PE-labelled anti-mouse IgG antibody. Data are expressed as the mean fluorescence intensity (MFI) ±SD of the results obtained from two independent experiments.

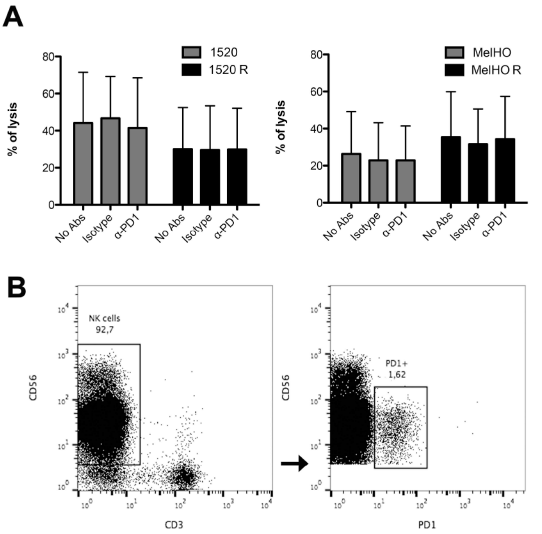

Supporting Information figure 2. NK cell recognition of BRAFi resistant variants after PD-1 blocking. (A) Standard Chromium release assay was performed following NK cells treatment for one hour with α-PD1 antibody (clone EH12.2H7) prior to co-incubation with 1520 and MelHO parental (grey columns) and 1520 and MelHO resistant (black columns). The data are displayed as the mean ±SD of three independent experiments. **(B)** Representative FACS plot of purified NK cells surface stained with anti-CD3 (clone UCHT1), anti-CD56 (clone HCD56) and same PD1 antibody used in the blocking experiments (clone EH12.2H7) detected by PE-labelled anti-mouse IgG antibody.

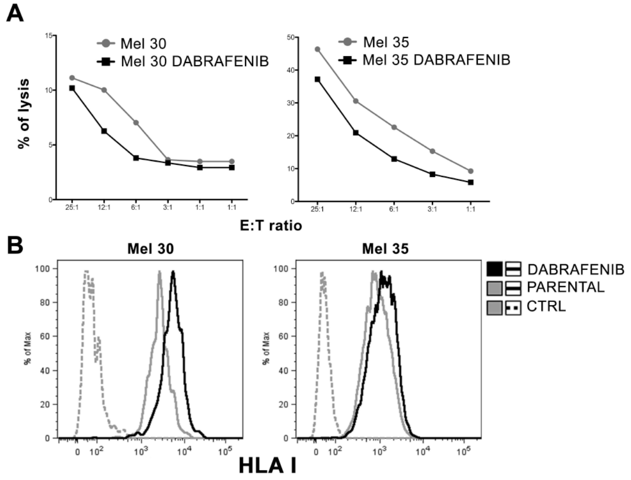

Supportin Information Figure 3. Acute treatment effect of dabrafenib on NK cells susceptibility. (A) Primary melanoma cells (Mel 30 and Mel 35) were treated with Dabrafenib for 24 hours and the cells were then tested for their susceptibility to NK cell killing in a standard chromium release assay. **(B)** FACS histograms showing HLA class I surface expression levels on Mel 30 and Mel 35 after 24 hours treatment with Dabrafenib. Data are representative of the results obtained from two independent experiments.

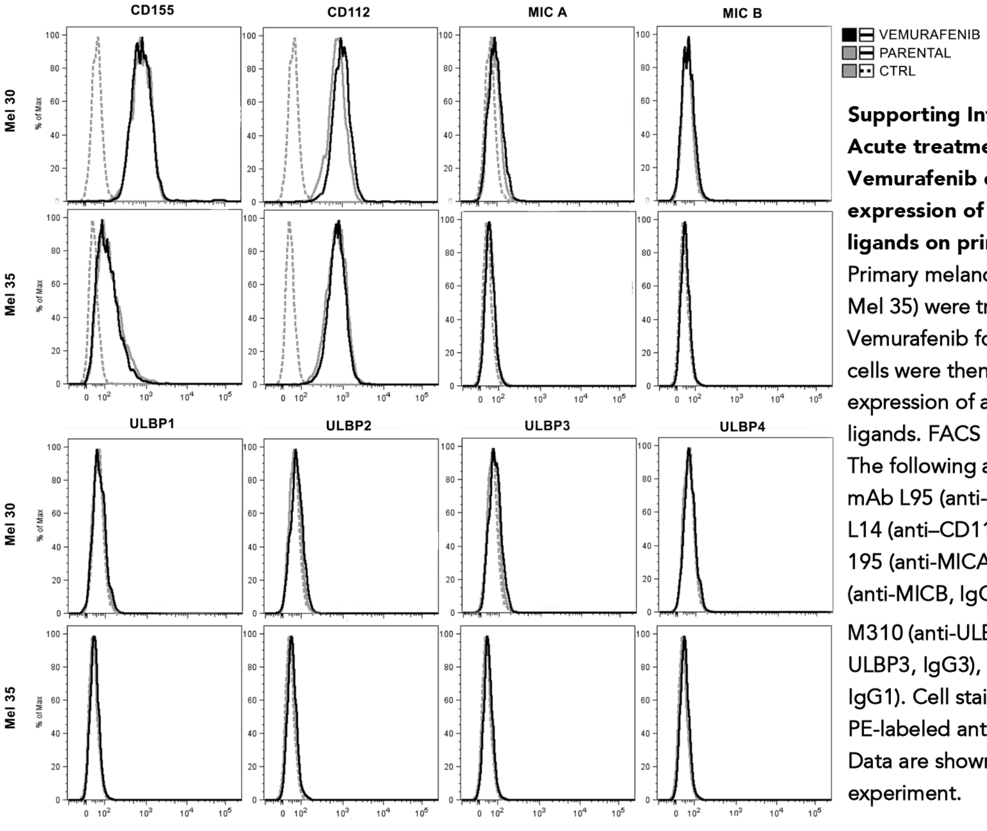

Supporting Information Figure 4. Acute treatment effect of Vemurafenib on the surface expression of NK cells activa ting ligands on primary melanoma cells. Primary melanoma cells (Mel 30 and Mel 35) were treated with Vemurafenib for 24 hours and the cells were then tested for the surface expression of activating NK cell ligands. FACS histograms are shown. The following antibodies were used: mAb L95 (anti-CD155, IgG1),mAb L14 (anti–CD112, IgG2a), mAb BAM 195 (anti-MICA, IgG1) and mAb6D4 (anti-MICB, IgG1), M295 (anti-ULBP1,IgG1) M310 (anti-ULBP2, IgG1),M550 (anti-ULBP3, IgG3), and M478 (anti-ULBP4, IgG1). Cell staining was detected by PE-labeled anti-mouse IgG antibody. Data are shown from one single experiment.

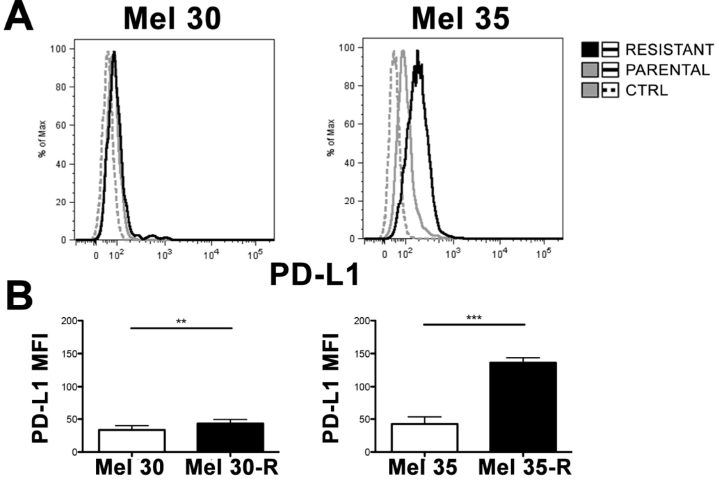

Supporting Information Figure 5. PDL-1 expression on BRAFi sensitive and resitant melanoma primary cells. (A) Primary melanoma cells were treated for 4 weeks with vemurafenib and then stained with anti-PDL1 (clone 10F.9G2). Representative FACS histograms for Mel 30 and Mel 35 where parental (gray) and resistant (black) and isotype (dashed) are included. (B) Bar charts show the mean ±SD of three independent experiments. Mel 30 $p=0.0042$, Mel 35 $p=0.0006$, paired student t-test.

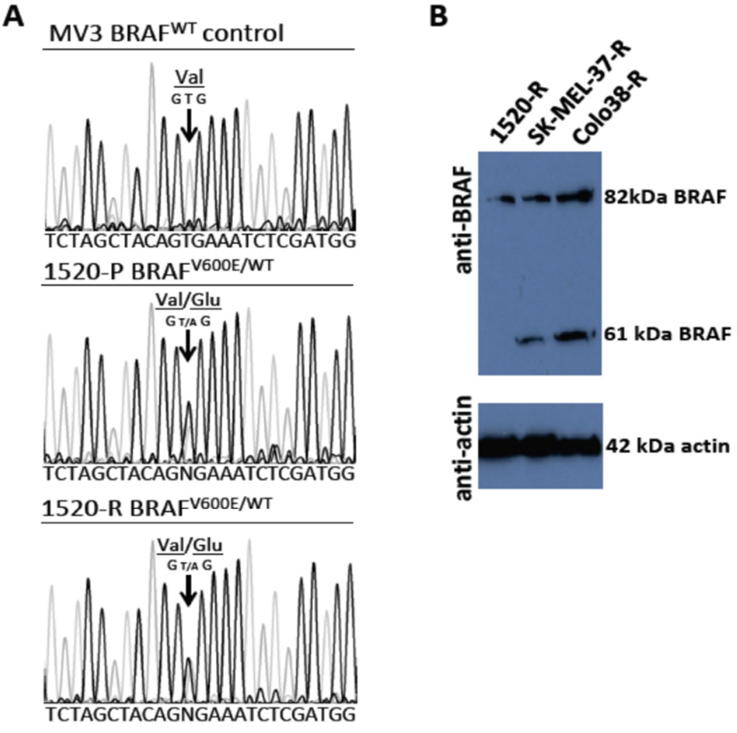

Supporting Information Figure 6. BRAF gene sequencing and BRAF protein detection in melanoma cell lines. A) BRAF exon15 region sequencing of MV3, 1520 and 1520-R cells. Chromatogram and base calls confirm absence of mutant BRAFV600E sequence in MV3 and indicate the presence of the mutant BRAFV600E allele in 1520 and 1520-R cells; **B)** Immunoblot detection of BRAF protein in lysates from 1520-R, SK-MEL37-R and COLO38-R cell lines. Membrane was reprobed with anti-actin antibody.

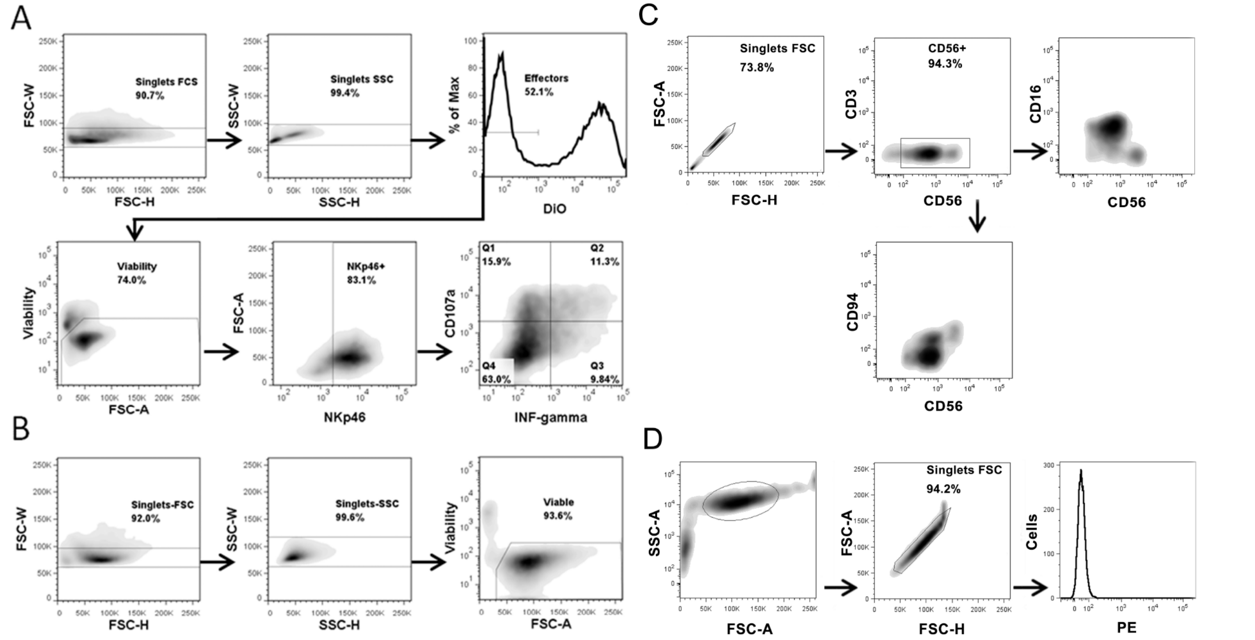

Supporting Information Figure 7. Flow cytometry gating strategy used. (A) NK degranulation and cytokine responce assay, (B) NKR ligand expression profiling, (C) NK cell phenotype evaluation and (D) tumor cells phenotype. A) Cell multiplets were excluded by FSC-H vs. FSC-W and SSC-H vs SSC-W gating, following by exclusion of DiO positive target cells, dead cells and NKp46 negative non-NK cells. B) cells multiplets were excluded by FSC-H vs FSC-W and SSC-H vs SSC-W gating, following exclusion of Hoechst positive dead cells. C) cell multiplets were excluded by FSC-H vs FSC-A gating, following by gating on CD3-CD56+ NK cells. D) Cells were selected using SSC-A vs FSC-A gating following cells multiplets exclusion by FSC-H vs FSC-A gating.
