# Supplementary material for: HLA class I downregulation is associated with enhanced NK‐cell killing of melanoma cells with acquired drug resistance to BRAF inhibitors
Source: Eur J Immunol. 2015 Dec 20;46(2):409–19. doi: 10.1002/eji.201445289 (PMC4832274; doi:10.1002/eji.201445289)
Supplement: Supplementary file 2 — Peer review correspondence [file EJI-46-409-s002.pdf]

Peer review correspondence

European Journal of Immunology

HLA class I downregulation is associated with enhanced NK-cell killing of melanoma cells with acquired drug resistance to BRAF inhibitors

Rosa Sottile, Pradeepa N. Pangigadde, Thomas Tan, Andrea Anichini, Francesco Sabbatino, Francesca Trecroci, Elvira Favoino, Laura Orgiano, James Roberts, Soldano Ferrone, Klas Kärre, Francesco Colucci and Ennio Carbone

Corresponding author: Ennio Carbone, Experimental and Clinical Medicine, Università degli Studi di Catanzaro "Magna Graecia", Germaneto, Catanzaro, Italy

---

Review Timeline:	Submission date:	24 October 2014
	First editorial decision:	9 December 2014
	First revision received:	24 June 2015
	Second editorial decision:	10 August 2015
	Second revision received:	22 September 2015
	Accepted:	5 November 2015

Handling Executive Committee members: Prof. Marco Colonna

Please note that the correspondence below does not include the standard editorial instructions regarding preparation and submission of revised manuscripts, only the scientific revisions requested and addressed.

First Editorial Decision – 9 December 2014

Dear Dr. Colucci,

Manuscript ID eji.201445289 entitled "Human NK cells target melanoma cells that have acquired drug resistance to targeted therapy in vitro" which you submitted to the European Journal of Immunology has been reviewed. The comments of the referees are included at the bottom of this letter.

The reviewers have many concerns and a revised paper should thoroughly address those critiques. The revised paper will be reviewed by the same reviewers. If the authors think that this cannot be achieved in a reasonable time, then we would encourage submission to another journal.

A revised version of your manuscript that takes into account the comments of the referee(s) will be reconsidered for publication.

You should also pay close attention to the editorial comments included below. *In particular, please edit your figure legends to follow Journal standards as outlined in the editorial comments. Failure to do this will result in delays in the re-review process.*

Please note that submitting a revision of your manuscript does not guarantee eventual acceptance, and that your revision will be re-reviewed by the referee(s) before a decision is rendered.

If the revision of the paper is expected to take more than three months, please inform the editorial office. Revisions taking longer than six months may be assessed by new referee(s) to ensure the relevance and timeliness of the data.

Once again, thank you for submitting your manuscript to European Journal of Immunology and we look forward to receiving your revision.

Yours sincerely,
Katharina Schmidt

On behalf of Prof. Marco Colonna

Dr. Katharina Schmidt
Editorial Office
European Journal of Immunology
e-mail: ejied@wiley.com
www.eji-journal.eu

****

Reviewer: 1

Comments to the Author

In this manuscript by Tan et al, the authors investigate the effects of acquisition of resistance to BRAF inhibitors by melanoma cells on their susceptibility to NK cell killing. The authors present data showing increased susceptibility to NK cell killing of one cell line, decreased susceptibility in another line, and no change in the remaining 2 cell lines. The final figure demonstrates decreased Class I in the line that was more susceptible. There are also some modest changes in PD-L1 and MIC-A/B. Overall, while this is an interesting concept, the current manuscript is underdeveloped and lacks any kind of mechanistic studies

European Journal of Immunology

of increased susceptibility to NK cell killing (or resistance for the 1520 line) or link between BRAFi resistance changes in NK cell ligands.

Reviewer: 2

Comments to the Author

The current study attempts to demonstrate the susceptibility of drug-resistant tumor lines to NK cell killing in comparison with untreated parental lines. They demonstrate one line (Mel Ho) has increased killing opf the resistant line whereas another line 1520 actually has lesser killing. Furthermore, other lines assessed had equivocal effects. They attempt to discern the mechanism underlying these effects with surface expression of various targets/ligands with subtle changes being observed but no real clear indication as to why differential killing effects are occurring. While potentially of interest, the variability (even opposite effects) observed with the cell lines make definitive conclusions difficult to extrapolate as may be cell line contingent. The effects in Figure 4 are also difficult to correlate as definitive blocking studies are needed to show they do play a role. It is unclear as to why higher killing in the resistant Mel Ho line is associated with lower MIC-A and why this was not assessed in 1520 or other lines. Figure 3 is key yet hard to discern based on significance and variability.

Reviewer: 3

Comments to the Author

Summary:

The article by Tan et al. evaluates the ability of IL-2 activated NK cells to respond to BRAF-inhibitor resistant melanoma cell lines. The potential for these studies has a high level of clinical relevance since resistance to BRAF inhibition in patients is a common long-term problem. The authors nicely demonstrated the generation of multiple resistant cell lines, and validated the V600E mutation that existed in one of the parent lines was maintained in the resistant cell line. The purpose of the report was to determine if the BRAF-inhibitor resistant cell lines would be sensitive to IL-2 activated NK cell killing. While the data presented has important clinical ramifications, additional studies are needed to confirm the ability of IL-2 activated NK cells to work in the pre-clinical setting.

Specific comments:

1. The methods section indicates that both the BRAF resistant and sensitive cell lines were cultured for two weeks prior to functional studies in inhibitor-free media. Did the tumor cells maintain their resistance after two weeks without exposure to the inhibitor? If all of the data presented are from after the two-week wash out period, it would be interesting to determine if the effect on NK cell sensitivity would be stronger

European Journal of Immunology

immediately following BRAF-inhibitor treatment. As seen with other agents, continued treatment with the inhibitor while ineffective at controlling tumor growth directly, may continue to sensitize the tumor cells to NK cell mediated killing.

2. Studies evaluating NK ligand expression on the parent and resistant cell lines indicated a potential mechanism for the differences in susceptibility to NK killing. These studies should be supported by additional in vitro confirmatory studies with blocking antibodies to determine the ligand interaction(s) that are responsible.

3. All of these studies were conducted with tumor cell lines, and while this suggests NK cells would still recognize and kill tumor targets, there is no primary data to support this. The manuscript can be strengthened by the addition of primary samples (BRAF sensitive and resistant) to validate they are sensitive to IL-2 activated NK killing.

4. There is no flow cytometry data in the main paper demonstrating the gating strategy for the functional studies. This is shown in Supplementary Figure 3. This figure should be included in the manuscript, along with the bar graph already depicted of the results.

5. The use of NKp46 as the single defining NK-specific marker is not standard. Including a flow cytometry stain of the IL-2 activated NK cells that includes CD56, CD3, CD94, and CD16 at a minimum would be helpful to evaluate the phenotype of the NK cells post culture, at the time of the functional assays.

<u>First revision – authors' response – 24 June 2015</u>

Reviewer: 1

Comments to the Author

In this manuscript by Tan et al, the authors investigate the effects of acquisition of resistance to BRAF inhibitors by melanoma cells on their susceptibility to NK cell killing. The authors present data showing increased susceptibility to NK cell killing of one cell line, decreased susceptibility in another line, and no change in the remaining 2 cell lines. The final figure demonstrates decreased Class I in the line that was more susceptible. There are also some modest changes in PD-L1 and MIC-A/B. Overall, while this is an interesting concept, the current manuscript is underdeveloped and lacks any kind of mechanistic studies of increased susceptibility to NK cell killing (or resistance for the 1520 line) or link between BRAFi resistance changes in NK cell ligands.

As requested, we have performed blocking experiments in which we mask MHC class I molecules on parental and resistant 1520 cells. Moreover the NK recognition of BRAFi melanoma variants have been further dissected by blocking activating receptors DNAM-1 and NKG2D. The new data set is shown in figure 5 (refers to paragraph "Immunomodulatory effects of BRAFi and MHC class I molecules on NK cell cytotoxicity", page 6 lines 22-26 and page 7 lines 1-2). We have also evaluated both the acute and the

chronic effect of BRAFi on primary melanoma cells (paragraph “Susceptibility of primary melanoma cells to NK cell cytotoxicity upon short-term BRAFi treatment and upon established BRAFi resistance” page 7-8, figure 6,7,8,9 and Supplementary 4). We show that acute treatment (24 hours) with both Vemurafenib (figure 6) and Dabrafenib (supplementary figure 4) decreased the susceptibility to NK cell killing of melanoma cells, due to a marked upregulation of MHC class I surface expression levels (figure 7 and supplementary figure 4). Upon generation of BRAFi resistant variants we observed a significant increased in NK cell susceptibility for one patient (Mel 30, figure 8), like the pattern observed for MEL-HO-R cell lines, (figure 3). The resistant variant from patient 2 (Mel 35) was still sensitive to NK cell mediated killing but at the same levels of the parental cells (figure 8), like the observation on COLO-38 cell lines (figure 3). The new experimental evidence confirms the inverse correlation between MHC class I expression at the cell surface and susceptibility to NK-cell mediated killing.

Reviewer: 2

Comments to the Author
The current study attempts to demonstrate the susceptibility of drug-resistant tumor lines to NK cell killing in comparison with untreated parental lines. They demonstrate one line (Mel Ho) has increased killing opf the resistant line whereas another line 1520 actually has lesser killing. Furthermore, other lines assessed had equivocal effects. They attempt to discern the mechanism underlying these effects with surface
expression of various targets/ligands with subtle changes being observed
but no real clear indication as to why differential killing effects are
occurring. While potentially of interest, the variability (even opposite effects) observed with the cell lines make definitive conclusions difficult to extrapolate as may be cell line contingent. The effects in Figure 4 are also difficult to correlate as definitive blocking studies are needed to show they do play a role. It is unclear as to why higher killing in the resistant Mel Ho line is associated with lower MIC-A and why this was not assessed in 1520 or other lines. Figure 3 is key yet hard to discern based on significance and variability.

As suggested by this reviewer we performed blocking studies to determine the molecular mechanism regulating the NK cytotoxic recognition of BRAFi resistant melanoma variants. As reported in the new figure 5 (refers to paragraph “Immunomodulatory effects of BRAFi and MHC class I molecules on NK cell cytotoxicity”, page 6 lines 22-26 and page 7 lines 1-2) we blocked the interaction between CD155 and CD112 with their receptor (DNAM-1) and the MIC A/B, ULBP 1-2-3-4 receptor (NKG2D) using monoclonal antibodies. Our data clearly showed that these ligands are not involved in the recognition of 1520R. Instead by masking MHC class I molecules we had the formal proof that MHC class I is the key regulatory mechanism in the regulation of the NK cell recognition of BRAFi resistant melanoma variants. Our new

European Journal of Immunology

data clearly indicate that the reduced expression of MHC class I on MEL-HO cells is the mechanism by which these cells have higher sensitivity to NK cell mediated killing after acquisition of drug resistance.

Reviewer: 3

Comments to the Author

Summary:

The article by Tan et al. evaluates the ability of IL-2 activated NK cells to respond to BRAF-inhibitor resistant melanoma cell lines. The potential for these studies has a high level of clinical relevance since resistance to BRAF inhibition in patients is a common long-term problem. The authors nicely demonstrated the generation of multiple resistant cell lines, and validated the V600E mutation that existed in one of the parent lines was maintained in the resistant cell line. The purpose of the report was to determine if the BRAF-inhibitor resistant cell lines would be sensitive to IL-2 activated NK cell killing. While the data presented has important clinical ramifications, additional studies are needed to confirm the ability of IL-2 activated NK cells to work in the pre-clinical setting.

Specific comments:

1. The methods section indicates that both the BRAF resistant and sensitive cell lines were cultured for two weeks prior to functional studies in inhibitor-free media. Did the tumor cells maintain their resistance after two weeks without exposure to the inhibitor? If all of the data presented are from after the two-week wash out period, it would be interesting to determine if the effect on NK cell sensitivity would be stronger immediately following BRAF-inhibitor treatment. As seen with other agents, continued treatment with the inhibitor while ineffective at controlling tumor growth directly, may continue to sensitize the tumor cells to NK cell mediated killing.
2. Studies evaluating NK ligand expression on the parent and resistant cell lines indicated a potential mechanism for the differences in susceptibility to NK killing. These studies should be supported by additional in vitro confirmatory studies with blocking antibodies to determine the ligand interaction(s) that are responsible.
3. All of these studies were conducted with tumor cell lines, and while this suggests NK cells would still recognize and kill tumor targets, there is no primary data to support this. The manuscript can be strengthened by the addition of primary samples (BRAF sensitive and resistant) to validate they are sensitive to IL-2 activated NK killing.
4. There is no flow cytometry data in the main paper demonstrating the gating strategy for the functional studies. This is shown in Supplementary Figure 3. This figure should be included in the manuscript, along with the bar graph already depicted of the results.

European Journal of Immunology

5. The use of NKp46 as the single defining NK-specific marker is not standard. Including a flow cytometry stain of the IL-2 activated NK cells that includes CD56, CD3, CD94, and CD16 at a minimum would be helpful to evaluate the phenotype of the NK cells post culture, at the time of the functional assays.

1. The primary cells used were maintained in the presence of the inhibitors without wash out period after generation of the resistant variants. For the specific methods refer to paragraph “Generation of BRAFi-resistant cells” page 14, lines 1-4. As suggested by this reviewer the effect on NK cell sensitivity immediately following BRAF-inhibitor treatment has been analysed. The new data are included in the paragraph “Susceptibility of primary melanoma cells to NK cell cytotoxicity upon short-term BRAFi treatment and upon established BRAFi resistance” page 7-8, figure 6,7,8,9 and Supplementary 4. We showed that acute treatment (24 hours) with both Vemurafenib (figure 6) and Dabrafenib (supplementary figure 4) decreased the susceptibility to NK cell killing of the melanoma cells. This reduction in NK susceptibility inversely correlate with increased levels of MHC class I surface expression (figure 7).
2. AS requested, and to clarify the mechanism leading to differences in NK cell susceptibility of the BRAFi resistant variant we performed blocking experiments using as model the 1520 and 1520R melanoma cell lines now included in the new figure 5 (refer to paragraph “Immunomodulatory effects of BRAFi and MHC class I molecules on NK cell cytotoxicity”, page 6 lines 22-26 and page 7 lines 1-2). Our data demonstrated that MHC class I play a major role in the recognition of the resistant variants while ruling out the potential involvement of the activating NK cell ligands investigated.
3. In the revised manuscript new data obtained from two primary melanoma cell lines (Mel 30 and Mel 35) have been added to address reviewer 3’s request. Using these freshly established primary cells we have validated the sensitivity to IL-2 activated NK cell killing of the BRAFi sensitive and resistant variants.
4. Please find the gating strategy description to supplementary figure 1. Noteworthy the new figure contains an extended evaluation of NK cells phenotype used in the functional assays as well as the gating strategy used for the evaluation of the tumor cells phenotype.
5. We have included in supplementary figure 1 the evaluation of NK cells phenotype that we used in the functional assays. Markers include CD3, CD56, CD94 and CD16.

<u>Second Editorial Decision – 10 August 2015</u>

Dear Dr. Carbone,

Please accept my sincere apologies for the delay in processing the re-review of your revised manuscript ID eji.201445289.R1 entitled "MHC Class I molecules on melanoma cells with acquired drug resistance to BRAF inhibitors regulate NK cell cytotoxicity" which you had submitted to the European Journal of Immunology. There was a difference in opinions among the reports for which additional advice from the

Executive Editor was sought. All opinions have now been assessed and the comments of the referees are included at the bottom of this letter.

You will see that Referee 2 was not satisfied with the revisions made; this referee felt that the subtle differences in some of your figures and the lack of a clear mechanism from this phenomenon warranted rejection. However the Executive Editor feels that a revision is feasible, and asks that you experimentally address the concerns of Referees 1 and 3 and discuss the weakness/limitations of your work in response to Referee 2 in your next revision. Please note that the journal does not encourage multiple rounds of revision and you should fully address the concerns of the referee in this final round of revision. Should you disagree with any of the referees' concerns, you should address this in your point-by-point response and provide solid scientific reasons for why you will not make the requested changes.

You should also pay close attention to the editorial comments included below. *In particular, please edit your figure legends to follow Journal standards as outlined in the editorial comments. Failure to do this will result in delays in the re-review process.*

Please note that submitting a revision of your manuscript does not guarantee eventual acceptance, and that your revision will be re-reviewed by the referees before a decision is rendered.

If the revision of the paper is expected to take more than three months, please inform the editorial office. Revisions taking longer than six months may be assessed by new referee(s) to ensure the relevance and timeliness of the data.

Once again, thank you for submitting your manuscript to European Journal of Immunology and we look forward to receiving your revision.

Yours sincerely,
Karen Chu

On behalf of Prof. Marco Colonna

Dr. Karen Chu
Editorial Office
European Journal of Immunology
e-mail: ejied@wiley.com
www.eji-journal.eu
****

European Journal of Immunology

Peer review correspondence

Reviewer: 1

Comments to the Author

This manuscript by Sottile et al has been greatly strengthened by the addition of new experiments and a focus on changes in MHC-class I on BRAF-R cells.

Specific comments:

1. The authors show that inhibition of Class I on 1520-R cells increases susceptibility to NK cell killing. However, it is not clear that this is the only mechanism of resistance to NK cell killing since the parental line also becomes more NK-sensitive (as would be expected with blocking class I). Is there a significant difference between % lysis of parental and 1520-R cells with anti-HLA-I (Figure 4B), as there appears to be? If so – the authors should discuss that this might not be the sole mechanism of NK resistance in this cell line.

2. On page 32, the authors state “Similar results were obtained using dabrafenib (Supplement 2)”, but are discussing increased MHC-I expression. Supplemental Figure 2 shows killing, but no data for Class I expression – do the authors have this data and if so it should be shown. If this data is not available the discussion should be changed accordingly.

3. The statistics for MHC-I expression in Mel30-R and Mel35-R cells show a “significant” difference, but do the authors think this is a biologically significant difference? The results are based on MFI which are shown as % of control, but the histograms in Figure 6 show very little difference (especially when compared with changes in Class I in other figures, such as Figure 5B). There are large differences in NK cell killing of Mel30 versus Mel30-R cells, and based on the flow shown it is hard to believe that this is due to Class I alone. This is worth a discussion and a more fair/balanced discussion of the data.

Reviewer: 2

Comments to the Author

This is a revised study attempting to address issues related to drug resistance in melanoma as it relates to NK cell killing capability. A primary issue is the marginal differences observed both in MFI changes and killing extent in the lines, inconsistency with regard to overall outcome and lack of clear mechanism due to variable and partial effects especially in figure 5 and 6.

Reviewer: 3

European Journal of Immunology

Comments to the Author
The revisions made thus far to the manuscript by Tan et al. have improved both the quality and the relevance of the data with regards to the effectiveness of NK cell-based therapy against BRAF resistant melanoma cells. However, the authors failed to evaluate a critical interaction that was found to be altered in the sensitive and resistant cell lines. Although the importance of PD-L1 expression was discussed in the paper, the authors failed to attempt blocking this interaction in the resistant lines to determine if this ligand also played a role in sensitizing BRAF resistant cells to NK killing, although it appears to be the most significantly dysregulated ligand. The potential impact of the manuscript on designing effective immunotherapies against resistant melanoma samples would be enhanced if these studies were added. In addition, the primary samples should be evaluated for PD-L1 expression as well given the results with the melanoma cell lines.

Second revision – authors' response – 22 September 2015

We agree with the comments and, accordingly, we now present what we feel is a more balanced view. Specifically, we have:
1) Changed the title by shifting the emphasis on the main discovery of the study, which is the susceptibility of both BRAFi-responsive and -resistant melanoma cells to NK cell cytotoxicity.
2) Discussed the role of MHC class I in the context of other potential interpretations.
Moreover, as suggested by Reviewer 1 and 3 and by the Editor, we have extended our analysis of the mechanisms to the PD1/PD1L pathway. The new data included in the revised manuscript clearly rule out this pathway in regulating the NK – melanoma cells cross talk. We now state this in the text of the manuscript.

Third Editorial Decision – 7 October 2015

Dear Dr. Carbone,

It is a pleasure to provisionally accept your manuscript entitled "NK cell targeting of melanoma cells with acquired drug resistance to BRAF inhibitors" for publication in the European Journal of Immunology. For final acceptance, please follow the instructions below and return the requested items as soon as possible as we cannot process your manuscript further until all items listed below are dealt with.

Please note that EJI articles are now published online a few days after final acceptance (see Accepted Articles: http://onlinelibrary.wiley.com/journal/10.1002/(ISSN)1521-4141/accepted). The files used for the

Peer review correspondence

Accepted Articles are the final files and information supplied by you in Manuscript Central. You should therefore check that all the information (including author names) is correct as changes will NOT be permitted until the proofs stage.

We look forward to hearing from you and thank you for submitting your manuscript to the European Journal of Immunology.

Yours sincerely,
Karen Chu

on behalf of Prof. Marco Colonna

Dr. Karen Chu
Editorial Office
European Journal of Immunology
e-mail: ejied@wiley.com
www.eji-journal.eu
